# Knowledge, Attitudes, and Practices of the Mexican Population Regarding the Disposal of Medications: A Cross-Sectional Study

**DOI:** 10.3390/epidemiologia7020044

**Published:** 2026-03-31

**Authors:** Raymundo Escutia-Gutiérrez, Igor Martin Ramos-Herrera, Anahí Dreser-Mansilla, Nelson Bruno de Almeida-Cunha

**Affiliations:** 1 Departamento de Farmacobiología, Centro Universitario de Ciencias Exactas e Ingenierías (CUCEI), Universidad de Guadalajara, Guadalajara 44430, Mexico; raymundo.escutia@academicos.udg.mx; 2 Departamento de Salud Pública, Centro Universitario de Ciencias de la Salud (CUCS), Universidad de Guadalajara, Guadalajara 44340, Mexico; igor.ramos@academicos.udg.mx; 3 Centro de Investigación en Sistemas de Salud, Instituto Nacional de Salud Pública, Cuernavaca 62100, Mexico; anahi.dreser@insp.mx

**Keywords:** medication, knowledge, attitudes and practices in health, medical waste disposal, One Health, Mexico

## Abstract

Background and Objectives: The improper disposal of expired and unused medications (EUM) poses significant environmental and health risks. Discarding EUM in household trash or drains leads to accidental poisoning, illegal trade, and ecosystem contamination. These persistent compounds often resist wastewater treatment, disrupting ecological balance and contributing to antimicrobial resistance, thereby increasing morbidity and mortality rates. This study aims to analyze the knowledge, attitudes and practices (KAP) and related factors of the Mexican population regarding the disposal of EUM. Methods: A cross-sectional, descriptive, and correlational study was conducted via an online survey of adults (18+) from October 2021 to October 2024. Results: Among 6080 participants (95.4% aged 18–59; 65.8% women), a medium level of KAP was observed. Notably, 51.5% did not use specialized disposal containers, only 15.5% knew container locations, and 30.5% correctly identified expiration dates. Significant associations emerged: lower education levels correlated with poorer disposal knowledge, while health-related backgrounds and postgraduate studies linked to positive attitudes and adequate practices. Ordinal logistic regression revealed that being elderly, belonging to a high socioeconomic class, having lower education levels, and lacking health-related studies were significantly associated with poor KAP regarding EUM disposal. Conclusions: Inadequate pharmaceutical disposal in Mexico compromises environmental and public health. Addressing this requires reinforced regulations, professionalized pharmacies, and a comprehensive approach to bridge knowledge gaps. Integrating digital tools—like real-time mapping and QR labeling—with accessible take-back schemes is vital in mitigating hazards and uphold the One Health triad.

## 1. Introduction

Medications are valuable therapeutic tools. However, improper disposal of expired and unused medications (EUM) poses environmental risks with potential public health implications. It is well known that large quantities of expired and leftover medications accumulate in households and are often discarded in the trash. This not only impacts the environment but also creates additional health risks, such as accidental consumption by children or pets, or even reaching the black market. Moreover, some EUM are disposed of into household drains. Many pharmaceutical compounds resist conventional wastewater treatment, leading to environmental accumulation and the disruption of ecosystems. This persistence fuels antimicrobial resistance, making once-treatable infections difficult to manage. Today, bloodstream infections caused by multidrug-resistant organisms represent a critical challenge, resulting in significantly higher rates of morbidity and mortality [1,2,3].

To address this, the World Health Organization (WHO) has issued guidelines for the proper disposal of pharmaceutical products [4]. In Mexico, the General Law for the Prevention and Integral Management of Waste classifies them as hazardous waste [5].

According to current Mexican Pharmacopeia regulations, establishments dedicated to the sale and supply of medications must implement comprehensive pharmaceutical waste management. Consequently, expired medications must be identified, classified, and finally collected by companies authorized by the Ministry of the Environment and Natural Resources of Mexico (SEMARNAT) for proper disposal. This is typically achieved through incineration or co-processing and must include all primary and secondary packaging (blisters, bottles, caps, droppers, and boxes) to prevent environmental risk and illegal reuse [6].

To prevent the improper disposal of medications in households, several countries have implemented ‘take-back’ programs to collect unused or expired medications, often involving special collection containers. Typically, pharmaceutical companies and pharmacies fund these programs under the “polluter pays” principle. Examples include Spain’s SIGRE program and Colombia’s Punto Azul program [1]. In Mexico, the pharmaceutical industry has established and financed a private system, the “Sistema Nacional de Gestión de Residuos de Envases y Medicamentos” (SINGREM), which collects EUM through containers mainly located in community pharmacies [7]. However, these programs face several challenges, including weak regulatory enforcement and low public participation, partly due to limited access to collection points and insufficient information provided by healthcare professionals [5,8,9].

Globally, studies have been promoted to understand how and why people dispose of EUM in specific ways. Such evidence is crucial for developing effective interventions and policies to address inappropriate disposal practices and protect public health and the environment [10,11]. Knowledge, attitudes, and practices (KAP) studies are used to find out what people think, believe, and how they act in relation to a specific issue [12].

In the context of medication disposal, knowledge refers to the understanding of concepts related to EUM disposal, attitudes refer to predispositions toward appropriate or inappropriate behaviors, and practices refer to the actual actions taken to dispose of medications [11]. Knowledge typically shapes attitudes, which in turn influence behaviors. Positive attitudes promote appropriate practices, and accurate knowledge supports the formation of positive attitudes [13]. Despite the importance of KAP studies on drug disposal, information on drug disposal in Latin America remains limited [10].

Previous research in Mexico has primarily examined the legal framework and management practices surrounding the disposal of EUM, as well as the characteristics of the discarded products and their environmental impact. However, limited attention has been given to the perspective of users. This study seeks to address that gap by analyzing the knowledge, attitudes, and practices of the Mexican population regarding EUM disposal, by posing the following research questions: (1) What is the level of knowledge, attitudes, and practices regarding the final disposal of EUM among the Mexican population? (2) Which population groups are at greatest risk of poor KAP regarding the disposal of EUM?

## 2. Materials and Methods

### 2.1. Study Area and Design

A cross-sectional, descriptive, and correlational study was conducted between October 2021 to October 2024, targeting the Mexican population aged 18 years and older. An 18-item self-administered Google Forms survey, adapted from a previous study, was used to assess knowledge, attitudes, and practices (KAP) regarding EUM disposal [14].

The survey was validated by expert review and piloted with 319 participants. The study was approved by the Ethics and Research Committees of the Jalisco Institute of Mental Health. An information sheet was presented to the participants online before the survey instrument. Participants indicated their consent by proceeding with the survey.

Participants were selected through convenience sampling. The survey was distributed at academic and scientific events and through social media (Facebook, Twitter, WhatsApp). Only complete responses were accepted, and informed consent was obtained from all participants.

### 2.2. Data Analysis

Statistical analysis was performed using SPSS version 20 (IBM Corp., Armonk, NY, USA). Frequencies were calculated for sociodemographic and KAP variables and correspond to the number of responses out of the total N. Pearson’s Chi-square test was used to identify significant associations between KAP variables and sociodemographic factors. Ordinal logistic regression was performed in order to examine the relationship between the total score of the survey and relevant sociodemographic predictors. The level of statistical significance was set at (α) = 0.05.

Age groups were classified into three categories: youth (15–29 years), adulthood (30–59 years), and elderly (60+ years), following the criteria of the National Demographic Dynamics Survey [15].

Educational level was stratified by years of study into basic (0–9) and intermediate (10–12) levels, as well as by completion of studies for bachelor’s equivalent (high level) and postgraduate levels, respectively. The survey also included a question to identify participants with health-related studies.

The socioeconomic classification was established with the number of people working and residing at home (0–3 points), number of bathrooms (0–2), cars (0–2), and rooms in the residence (0–3), as well as availability of internet at home (0–1). A range of scores was obtained from 0 to 11, grouped into low (0–3), medium (4–7), and high (8–11) levels.

KAP levels were determined using scoring ranges and divided into low, medium, and high percentiles. Knowledge scores ranged from 0 to 14 (low: 0–3; medium: 4–7; high: 8–14). Attitude scores ranged from 6 to 30, with higher scores indicating more positive attitudes (low: 6–23; medium: 24–26; high: 27–30). Practice scores ranged from 0 to 18, with higher scores indicating better practices (low: 0–5; medium: 6–9; high: 10–18). For the total survey score, the range was 0–58, with low (0–36), medium (37–42), and high (43–58) levels.

## 3. Results

### 3.1. Sociodemographic Characteristics

Table 1 shows some sociodemographic aspects of the participants. A total of 6080 respondents completed the survey, of which 4001 (65.8%) were women, and 2079 (34.2%) resided in Jalisco. Most participants (95.4%) were under 60 years of age. Socioeconomic levels were high in 58.6% of participants, medium in 39.9%, and low in 1.5%. Regarding education, 4.2% had a basic level, 22.4% intermediate, 55.5% bachelor’s degree, and 17.9% postgraduate degree. More than half (54.8%) reported health-related studies.

### 3.2. Knowledge on EUM Disposal

Regarding knowledge, 69.7% of participants did not know how to correctly identify the expiration date of medications, and only 15.5% were aware of the SINGREM program or the location of its containers. Additionally, 48.2% were unaware of how to reduce the risks associated with EUM. Approximately half of the population (45.1%) stated that donating unused medications is an appropriate way to reduce or control the risks associated with expired or unused medications. More information related to knowledge on EUM disposal can be found in Table 2.

### 3.3. Attitudes Towards EUM Disposal

Most participants (81.4%) agreed that EUM posed potential household risks. Furthermore, 89.1% believed that collection programs should be mandatory in all Mexican pharmacies, and 89.0% agreed that health professionals should provide information on proper medication disposal. Almost all participants (90.8%) emphasized the importance of public participation in EUM management. Only 31.0% believed incentives were necessary to encourage proper disposal (Table 3).

### 3.4. Practices Related to EUM Disposal

**Figure 1:**
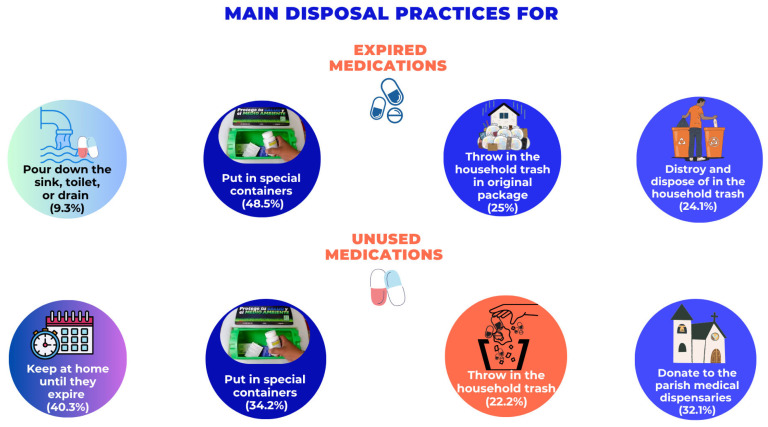
Percentage distribution of the main disposal practices for expired and unused medications in Mexico. Note: Questions with multiple answers that may exceed 100%.

Only 30% reported not having expired medications at home, while 49% kept them for potential future use. About 48% reported using special containers for expired medications, but a similar percentage disposed of them in household trash. These percentages were even lower for unused medications, which were mostly kept or donated (Figure 1).

Only 23.2% reported receiving information from doctors about proper medication disposal. Data concerning EUM disposal practices are detailed in Table 4.

### 3.5. Prediction of Sociodemographic Variables on the Disposal of Medicines

The ordinal logistic regression model examined the association between age group, gender, socioeconomic status, educational level, and health-related studies on the total score of the questionnaire of disposal of expired and unused medications. The model was statistically significant (χ^2^ = 372.950; *p* < 0.001) and explained 18.1% of the variance on medications disposal (Nagelkerke R^2^ = 0.181). No significant association was observed with sex (*p* = 0.112). Elderly populations showed a lower likelihood of proper disposal of expired and unused medications compared to youth and adulthood. Participants from the low or high socioeconomic classes had a lower likelihood of proper medication disposal compared to those from the medium socioeconomic class (OR = 1.15; 95% CI: 1.04–1.27; *p* = 0.005). Lower educational levels were associated with a lower probability of achieving a better questionnaire score. Additionally, not having health-related studies was associated with a lower likelihood of proper medication disposal (OR = 0.25; 95% CI: 0.22–0.28; *p* < 0.001). These and other parameters of the ordinal logistic regression related to predictors of socioeconomic variables on the disposal of medicines can be found in Table 5.

### 3.6. Associations Between Sociodemographic Variables and KAP

Significant associations were found between higher knowledge levels and age (χ^2^ = 45.988; *p* < 0.000), sex (χ^2^ = 7.699; *p* = 0.022), socioeconomic status (χ^2^ = 12.763; *p* = 0.012), education level (χ^2^ = 347.699; *p* < 0.000), and health-related studies (χ^2^ = 1042.284; *p* < 0.000).

**Figure 2:**
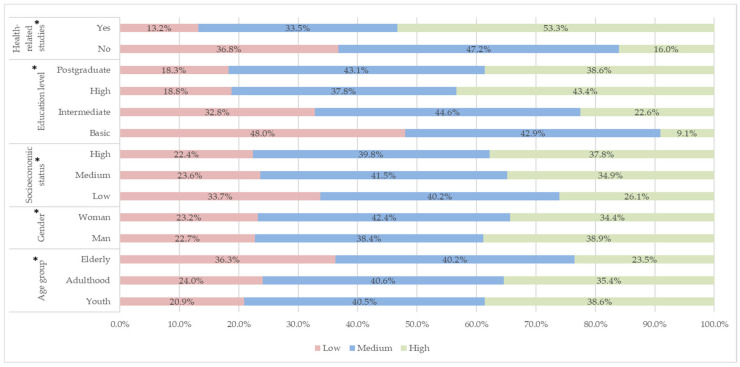
Relative percentages of each sociodemographic variable according to the level of knowledge. Note: * Statistically significant effect of the variables on the level of knowledge.

Figure 2 shows the relative percentages of each sociodemographic variable according to the level of knowledge. In relation to age range, it is observed that youth and adulthood (lower range) are associated with medium-to-high knowledge, while older adults have a predominance of low knowledge. Regarding the level of knowledge, it was found that the higher the socioeconomic level, the greater the knowledge about the management of EUM. Similarly, the lower the level of education, the lower the level of knowledge. People with a bachelor’s degree have a higher level of knowledge, even higher than those with a postgraduate degree. There is an association between belonging to the health area and having greater knowledge. The overall level of knowledge was mostly medium at 50.92% (3096), followed by high 26.03% (1583) and low 23.04% (1401). Adequate knowledge corresponds only to high levels.

The results indicate that there is a significant association between positive attitudes and age range (χ^2^ = 63.297; *p* < 0.000), sex (χ^2^ = 31.075; *p* < 0.000), level of education (χ^2^ = 156.703; *p* < 0.000), and belonging to the health area (χ^2^ = 205.293; *p* < 0.000), but there is no significant association with socioeconomic level (χ^2^ = 2.640; *p* = 0.620).

**Figure 3:**
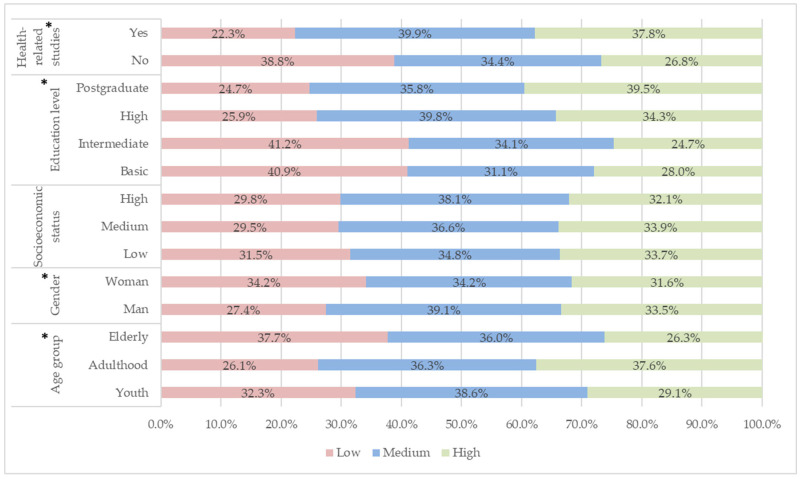
Relative percentages of each sociodemographic variable according to the level of attitudes. Note: * Statistically significant effect of the variables on the level of knowledge.

Figure 3 shows the relative percentages of each sociodemographic variable according to the level of attitudes. The lower age range, such as youth and, for the most part, adulthood, is associated with higher level of attitudes, while the elderly have a predominance of low level of attitudes. There is an association between female sex and positive attitudes about the management of EUM. With respect to schooling, participants with a lower level of education (basic and intermediate) have a lower level of attitudes about the final disposal of medications. People with postgraduate degrees had higher levels of attitudes than those with bachelor’s degrees. There is an association between belonging to the health area and having positive attitudes. The overall level of attitudes was mostly medium 37.45% (2277), followed by low 32.84% (1997) and high 29.70% (1806). Positive attitudes correspond only to high levels.

The results indicate that there is a significant relationship between the level of practices and socioeconomic level (χ^2^ = 6.312; *p* = 0.037), level of education (χ^2^ = 95.586; *p* < 0.000), and belonging to the health area (χ^2^ = 458.453; *p* < 0.000), but there is no significant association with age range (χ^2^ = 6.312; *p* = 0.177) or sex (χ^2^ = 2.859; *p* = 0.239).

**Figure 4:**
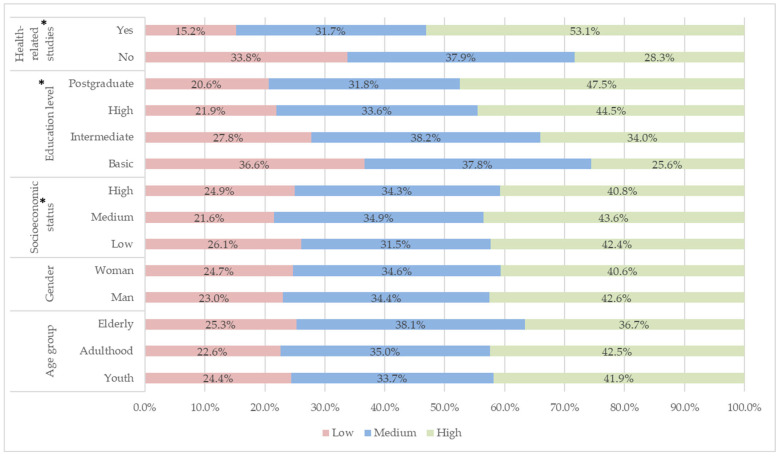
Relative percentages of each sociodemographic variable according to the level of practices on the final disposal of EUM. Note: * Statistically significant effect of the variables on the level of knowledge.

Figure 4 shows the relative percentages of each sociodemographic variable according to the level of practices on the final disposal of EUM. The lower age range, such as youth and, for the most part, adulthood, present adequate practices, while elderly have a predominance of inadequate practices. High levels of EUM management practices are associated with low and medium socioeconomic levels. With respect to education, the participants with the lowest level of education (basic and intermediate) presented inadequate practices in the disposal of medications. People with postgraduate and undergraduate degrees had adequate practices, and there was an association between belonging to the health area and having a high level of practice. The overall level of practice was mostly medium 45.83% (2787), followed by high 30.57% (1859) and low 23.58% (1434) levels. Appropriate practices correspond only to high levels.

## 4. Discussion

The objective of the study was to analyze the knowledge, attitudes, and practices of the Mexican population regarding the provision of EUM. Descriptive results indicated a predominant medium level across all KAP dimensions. An ordinal logistic regression model was applied, revealing that the elderly, individuals from high socioeconomic classes, those with lower education levels, and participants without health-related studies were significantly associated with a poorer KAP regarding EUM disposal. These findings suggest that while general awareness is moderate, specific demographic and educational barriers prevent the transition to optimal disposal behaviors. Our results are consistent with previous studies reporting that male sex, older age, lower educational level, and not being a healthcare professional significantly predict perceptions toward the improper disposal of EUM [16,17,18]. However, regarding participants’ socioeconomic status, our findings differ from those reported by Hajj et al. (2022) [17], who suggested that a higher socioeconomic level was associated with more appropriate disposal practices for expired or unused medicines. This discrepancy may be explained by differences in the operationalization of socioeconomic status. In our study, socioeconomic level was assessed using household characteristics (e.g., number of employed household members, number of residents, number of vehicles, and number of rooms), whereas Hajj et al. (2022) [17] and Akande-Sholabi et al. (2025) [16] relied primarily on monthly income as the main indicator. These methodological differences may account for the contrasting results observed.

Specifically, the desired thresholds—defined as high knowledge, positive attitudes, and adequate practices—were achieved by only 26.0%, 29.7%, and 30.6% of the sample, respectively. This consistency across the three dimensions contrasts with the “dissonance” reported by Lam and Hiew [10] as well as Low [11], in which high knowledge or positive attitudes coexist with predominantly inadequate practices. In our study, knowledge, attitudes, and practices were positively associated with higher educational levels and health-related studies.

Regarding knowledge, some key findings emerged. Most participants were aware of the risks associated with EUM, and nearly 80% recognized that the proper way to dispose of them is by using special collection containers. These findings are consistent with Lam’s 2024 study, which reported that slightly more than 70% of healthcare personnel and students were aware of appropriate medication disposal methods [10].

Despite this general awareness, less than half of the participants in our study were familiar with the SINGREM drug return system, and only 15% knew where the collection containers were located. This is particularly concerning given that the sample consisted mostly of individuals with high educational levels. In contrast, a previous survey of the general Mexican population found that 99% were unaware of SINGREM’s existence, and only 1.5% had seen drug collection campaigns [19].

Another important finding is that only 30.5% of respondents correctly understood that medications are valid until the last day of the month indicated on the packaging. This confusion may be linked to the current labeling practices in Mexico, where only the month and year of expiration are displayed, underscoring the need to update labeling regulations.

In terms of attitudes, most participants acknowledged that expired or unused medications pose potential risks in the home. A large majority also agreed that EUM collection programs should be mandatory for all pharmacies in Mexico and that the general public should actively participate in their proper disposal. Notably, 89% of respondents believed that health professionals should provide information on appropriate medication disposal, a finding consistent with Lam’s report [10], in which nearly 70% of the health professionals surveyed indicate a positive attitude about their responsibility to provide information to others about the proper disposal of EUM.

Concerning practices, a significant gap emerged. Although most participants knew that EUM should be disposed of in special containers, over half reported using inappropriate disposal methods, such as throwing medications in household garbage or pouring them down the drain. This reveals a clear disconnect between knowledge and actual practices, which may be explained by limited awareness of or poor access to SINGREM containers. In our study, 49.1% reported discarding EUM in household trash, and only 24% destroyed the medications before disposal, which could prevent their reuse.

These findings are consistent with two studies in African countries: Bekele et al. [20] found a high percentage of the respondents (76%) to be well informed on how to dispose of EUM, but most of them (95%) indicated that they would not be willing to be involved in EUM-take-back programs even if there had been such a mechanism. In the same vein, Esseku et al. [21] reported that 98% of participants dispose of their solid and semisolid medications with their general waste, and 81% dispose of liquid medications in the same way.

Similar disposal patterns have been reported in other studies conducted in Mexico and Latin America. The study conducted by Espinal and colleagues [19], in a general Mexican population, concludes that 70.7% choose to dispose of medications in the general garbage. A study conducted by Sanabria and contributors, in the Mexican city Tuxpan [22], reported that 65% of participants discarded medications in regular trash, 15% in the sewage system, and 3% burned them; moreover, none of the respondents were aware of local collection centers. Likewise, Quijano and colleagues [23] reported that 90% of participants in Bogotá, Colombia, disposed of medications with household waste, and 92% were unaware of collection sites for expired medications. The improper disposal practices pose a risk of EUM entering the environment or being subsequently used in falsification of medications [24,25]. These disposal methods introduce pharmaceuticals directly into the environmental media, causing a serious impact on environmental health, such as water pollution. Environmental health is part of what the World Health Organization called “One Health”, an integrated, unifying approach to balance and optimize the health of people, animals and ecosystems [26]. Continuous monitoring of EUM waste and its final destination is essential in mitigating this One Health issue—a Global Health concept that links human, animal, and environmental health. This framework is specifically used to study how pharmaceutical waste affects the planet.

Further analysis of practices revealed that nearly half of respondents reported storing expired medications at home. Half of the participants kept medications for potential future use, and about one-third cited medication accumulation due to surplus supply from healthcare services. This aligns with SINGREM’s 2024 statistics [27], which indicate that 66% of the collected medications originated from the public health sector, with paracetamol being the most frequently discarded drug [24]. Overprescribing not only contributes to the accumulation of medications at home but also significantly affects various areas of healthcare. It increases the risk of potentially inappropriate prescription, which is associated with higher rates of adverse effects, hospitalizations, and drug-related mortality [28].

Additionally, 20% of participants reported keeping medications because they felt it was wasteful to discard them, and 32% donated unused medications to dispensaries or religious organizations. Supporting this tendency, 45% believed that donating or sharing medications before they expire is a viable strategy to reduce EUM risks. Similar findings were reported in a Malaysian study [29], in which 68% of respondents preferred to donate unused medications rather than discard them, and approximately 50% were willing to share excess medications with others.

What are the implications for public health in Mexico? According to SINGREM’s statistics [27], 6625 tons of medications have been collected between 2010 and 2024, of which 618 tons were in 2024. However, based on our findings and those from previous studies reporting widespread retention and improper disposal of EUM [19,22], it is evident that significant environmental and public health risks persist.

This study highlights knowledge gaps regarding EUM disposal and emphasizes the need to strengthen public awareness through educational campaigns. It is equally important to improve the ability of health professionals to provide guidance on this issue. Considering that women are typically the primary managers of medications in households and that older adults are among the largest consumers of medications [30], these groups should be prioritized in communication strategies about the proper disposal of EUM.

Efforts to improve EUM disposal must be accompanied by accessible, affordable, and sustainable collection systems [11]. Currently, SINGREM operates only 4667 collection containers across 25 of the 32 states, covering roughly half of the country’s population. Many countries have successfully expanded medication collection through community pharmacies, which could be a feasible strategy for Mexico [9]. Expanding collection services would also capitalize on the positive public attitudes toward proper medication disposal and public participation identified in this study. However, our results also reveal a reluctance to discard unused medications and a tendency to seek donation options. Although dispensaries operated by civil or religious organizations already exist in Mexico, stronger regulation is needed to ensure safe and appropriate handling of donated medications.

Another key factor contributing to EUM accumulation is the overprescription and overproduction of medications, as reflected in our findings. This waste of medications presents not only an environmental and economic issue but also an ethical concern. Although collection systems reduce the risk of EUM misuse and environmental contamination, incineration—the current disposal method—also contributes to pollution. Therefore, it is essential to reduce the volume of collected and incinerated medications by improving prescribing and dispensing practices and considering strategies such as shelf-life extension programs [30].

Finally, the low identification rate of specialized containers (15.5%) underscores a failure in primary prevention. For effective epidemiological surveillance, interventions must focus not only on waste management logistics but also on addressing the behavioral risk factors (knowledge and attitudes) identified in this study to mitigate long-term health impacts.

Based on our findings, we propose four actionable pillars to improve expired and unused medications (EUM) management in Mexico:

1. Infrastructure Optimization: The SINGREM system should expand its reach by increasing the density of collection points in high-traffic hubs (e.g., supermarkets and public transport) and launching a real-time digital map to reduce geographical barriers.
2. Technological Integration: Transitioning from passive labeling to interactive QR codes on primary packaging. These should link directly to geo-located disposal sites and brief educational media, addressing the lack of awareness found in our study.
3. Regulation: Adopting mandatory ‘take-back’ programs for pharmacies or the implementation of secure 24 h disposal kiosks in pharmacies and medical services buildings, similar to the U.S. model. For rural settings, public health services should offer information and disposal containers. Additionally, explicit regulation on medicines donations should be developed.
4. Source Reduction: Promoting a shift toward unit-dose dispensing—a strategy proven effective in Spain—to prevent the initial accumulation of surplus medication in households. In the Mexican context, effectively mitigating the environmental and clinical risks associated with leftover medication requires prioritizing the professionalization of community pharmacies. Currently, most community pharmacies are operated by non-professionalized personnel rather than licensed pharmacists. Substantive improvement must be preceded by regulatory reforms that mandate the presence of qualified professionals capable of overseeing the integrity of the dispensing process.

The main limitations of this study include selection bias, as the sample predominantly comprised individuals with high socioeconomic and educational levels, a high proportion of healthcare workers, and an overrepresentation of women and younger adults. This profile restricts the generalizability of our findings to the broader Mexican population. Furthermore, since the survey was distributed online, the results likely reflect a ‘best-case scenario’ regarding knowledge and practices. This digital recruitment method inadvertently excludes a significant portion of the population affected by the digital divide in Mexico—85% or the population has access to the internet in urban areas vs. 60% in rural settings [31]. Consequently, the lack of awareness observed in this study is likely underestimated; such gaps are expected to be far more pronounced in rural areas or marginalized urban settlements, where limited internet connectivity, lower educational attainment, and reduced access to information technology further exacerbate health disparities.

## 5. Conclusions

While participants in this study demonstrated moderate levels of knowledge, attitudes, and practices regarding EUM disposal, more than half still employed inadequate disposal methods, posing risks to public health and the environment. The results also showed that older adults, individuals of higher socioeconomic status, those with lower education levels, and participants without health-related training were significantly associated with poorer KAP regarding EUM disposal. It is essential to update and enforce regulations regarding medication labeling, disposal, and donation, while simultaneously improving prescribing and dispensing practices.

Our analysis of behavioral determinants provides a robust foundation for evidence-based policy and interventions aimed at systemic risk mitigation. Specifically, addressing this public health challenge requires a multi-faceted strategy that integrates digital health tools—such as interactive QR codes and real-time mapping—with expanded infrastructure, including centralized collection points in high-traffic urban hubs. To advance these efforts, future research must identify specific barriers to utilizing national pharmaceutical take-back schemes, particularly within underserved rural settings. Furthermore, professionalizing community pharmacies by mandating the presence of licensed pharmacists is essential to oversee the dispensing lifecycle and implement unit-dose strategies aligned with successful international models. Ultimately, bridging knowledge gaps through targeted educational media and ensuring the availability of secure, accessible disposal services are vital to mitigating environmental hazards and uphold the One Health triad.

## Figures and Tables

**Table 1:** Sociodemographic characteristics of participants (*N* = 6080).

Variables	Frequency (%)
**Gender**	
Female	4001 (65.8)
Male	2079 (34.2)
**Age (years)**	
18–29	3017 (49.6)
30–59	2782 (45.8)
≥60	281 (4.6)
**Marital status**	
Single	3729 (61.3)
Married	1936 (31.9)
Divorced	337 (5.6)
Widowed	78 (1.3)
**Educational level**	
No formal education and basic level (0–8)	254 (4.2)
Middle (9–12)	1361 (22.4)
High	3375 (55.5)
Postgraduate	1090 (17.9)
**Health-related studies**	
Yes	3332 (54.8)
No	2748 (45.2)

**Table 2:** Knowledge on disposal of expired and unused medicines (***N*** = 6080).

Questions	Answers	Frequency (%)
From what day is a medication with an expiration date of March 2022 considered expired?	* 1 April 2022	1856 (30.5)
	1 March 2022	2471 (40.6)
	31 March 2022	1753 (28.9)
Do you understand the proper handling and disposal process for medicines and their packaging waste?	No	2833 (46.6)
	* Yes	3247 (53.4)
What is the most appropriate way for the final disposal of expired or unused medicines by the user?	* Placing them in special containers	4790 (78.8)
	Destroying them before throwing them in the household trash	492 (8.1)
	Burning them	69 (1.1)
	Throwing them in their original package into the household trash	492 (8.1)
	Pouring them down the sink, toilet, or drain	237 (3.9)
What are the possible effects of improper disposal of expired or unused drugs? **	* Environmental pollution	5042 (82.9)
	* Drug counterfeiting	3668 (60.3)
	* Drug toxicity	2779 (45.7)
	* Loss of drug efficacy	1462 (24.0)
	* Bacterial resistance	1795 (29.5)
	No effects	191 (3.1)
How can the risks associated with expired or unused medicines be reduced or controlled? **	* Providing consumers with proper guidance on their medications	1812 (29.8)
	* Placing expired medications in special containers	4589 (75.5)
	* Checking the expiration date of medications before using them	1654 (27.2)
	* Including information about final disposal on the label or box of the medication	3068 (50.5)
	* Prescribing doses, intervals, and durations that support therapeutic adherence	2464 (40.5)
	Increasing the amount of medications prescribed by the doctor	277 (4.6)
	Donating or sharing unused medications with others before they expire	2740 (45.1)
Do you know the “Sistema Nacional de Gestión de Residuos de Envases y Medicamentos” (SINGREM)?	No	3412 (56.1)
	Yes, but I do not know the location of the special containers	1725 (28.4)
	* Yes, and I also know the location of the special containers	943 (15.5)

Note: * Correct answers. ** Questions with multiple answers that may exceed 100%.

**Table 3:** Frequency and percentage of attitudes about the management of unused or expired medications (***N*** = 6080).

**Questions**	**Strongly Disagree** ***n*** **(%)**	**Disagree** ***n*** **(%)**	**Neutral** ***n*** **(%)**	**Agree** ***n*** **(%)**	**Strongly Agree** ***n*** **(%)**
1. Expired or unused medications can pose potential hazards in the home.	287 (4.7)	161 (2.6)	681 (11.2)	2064 (33.9)	2887 (47.5)
2. The media lacks adequate information on the proper disposal of medicines present in the home.	261 (4.3)	159 (2.6)	592 (9.7)	2228 (36.6)	2840 (46.7)
3. Health professionals should provide the general population with information on the proper disposal of medicines present in the home.	257 (4.2)	72 (1.2)	338 (5.6)	2075 (34.1)	3338 (54.9)
4. Collection programs for expired or unused medicines should be mandatory for all pharmacies in Mexico.	231 (3.8)	78 (1.3)	356 (5.9)	1769 (29.1)	3646 (60.0)
5. The general population should be actively involved in the proper disposal of medicines.	215 (3.5)	51 (0.8)	295 (4.9)	2134 (35.1)	3385 (55.7)
6. People who deposit expired or unused medicines in the special containers for this purpose deserve some kind of incentive, either financial or in kind.	659 (10.8)	1180 (19.4)	2358 (38.8)	1141 (18.8)	742 (12.2)

Note: Strongly agree and agree are considered positive attitudes.

**Table 4:** Practices related to EUM disposal (*N* = 6080).

Questions	Answers	Frequency (%)
Do you check the expiration date of medicines before use?	No	159 (2.6)
	*** Yes	5168 (85.0)
	At times	753 (12.4)
Do you currently have expired medications in your home?	*** No	1864 (30.6)
	Yes	2802 (46.1)
	I do not know	1414 (23.3)
How do you dispose of expired medicines? **	*** I place them in special containers	2949 (48.5)
	I destroy them before throwing them in the household trash	1466 (24.1)
	I burn them	130 (2.1)
	I do not dispose of them, because I do not know how to do it.	192 (3.2)
	I throw them in their original package into the household trash	1522 (25.0)
	I pour them down the sink, toilet, or drain	568 (9.3)
What do you do with the medications you no longer use? **	*** I place them in special containers	2077 (34.2)
	I donate them to the parish medical dispensaries	1953 (32.1)
	I keep them at home until they expire	2451 (40.3)
	I throw them in the household trash	1352 (22.2)
	I sell them	62 (1.0)
	I pour them down the sink, toilet or drain	231 (3.8)
What are the main reasons for the accumulation of unused medications in your home? **	I consider it a shame to waste medication	1246 (20.5)
	I don’t know where the special containers are located	2107 (34.7)
	*** The doctor told me to change my treatment	1858 (30.6)
	They dispense too much medication to me	1993 (32.8)
	*** I had an adverse reaction to the medication and stopped the treatment	1002 (16.5)
	In case I need the same medication in the future	2977 (49.0)
	I felt better and stopped taking the medication	2131 (35.0)
Where do you obtain information on the proper disposal of medicines? **	*** At the doctor’s office	1408 (23.2)
	*** In pharmacies	1452 (23.9)
	On the Internet (websites, social networks, mobile applications)	3134 (51.5)
	In the media (radio, television, written press)	519 (8.5)
	I do not have access to such information	1458 (24.0)
	By family and friends	1010 (16.6)

Note: ** Questions with multiple answers that may exceed 100%. *** Good practices.

**Table 5:** Ordinal logistic regression of sociodemographic predictors on the disposal of medications.

Predictor	Category	β	OR	95% CI	*p*-Value
Age	Youth vs. elderly	0.270	1.309	1.025–1.673	**0.031**
	Adulthood vs. elderly	0.497	1.644	1.296–2.086	**<0.001**
Sex	Male vs. female	−0.0083	0.920	0.831–1.019	0.112
Socioeconomic status	Low vs. high	0.009	1.009	0.677–1.504	0.965
	Medium vs. high	0.140	1.15	1.042–1.270	**0.005**
Educational level	Basic vs. postgraduate	−0.549	0.577	0.436–0.764	**<0.001**
	Intermediate vs. postgraduate	−0.398	0.672	0.564–0.799	**<0.001**
	High vs. postgraduate	0.139	1.149	0.994–1.327	0.058
Health-realated studies	No vs. yes	−1.395	0.248	0.223–0.276	**<0.001**

Note: β—Logistic regression coefficient; OR—odds ratio; CI—confidence interval. All reported *p*-values are statistically significant at *p* < 0.05.

## Data Availability

The data presented in this study are available on request from the corresponding author due to privacy and ethical restrictions.

## Abbreviations

The following abbreviations are used in this manuscript:

EUM	Expired and Unused Medications
KAP	Knowledge, Attitudes and Practices
SINGREM	Sistema Nacional de Gestión de Residuos de Envases y Medicamentos
WHO	World Health Organization
